# Negative elongation factor: a key factor in the maintenance of intestinal epithelial barrier integrity

**DOI:** 10.1038/s41423-021-00817-2

**Published:** 2022-01-07

**Authors:** Elif Gelmez, Andreas Jeron, Dunja Bruder

**Affiliations:** 1 Infection Immunology Group, Institute of Medical Microbiology, Infection Control and Prevention, Health Campus Immunology, Infectiology and Inflammation, Otto-von-Guericke University, Magdeburg, Germany; 2 Immune Regulation Group, Helmholtz Centre for Infection Research, Braunschweig, Germany

**Keywords:** Mucosal immunology, Inflammation

In addition to its physiological function in the uptake of dietary nutrients, the intestinal epithelium constitutes an essential mechanical barrier separating luminal gut content and mucosal microbiota from the inner body. Intestinal epithelial cells (IEC) comprise the frontline of this barrier, the maintenance of which critically depends on the expression of cell–cell junction protein structures, including tight junctions, adherence junctions, desmosomes, and gap junctions, which physically bridge and seal the intercellular niche within the IEC layer. Consequently, to fulfill their function as cellular gatekeepers in the gut, high basal transcriptional activity of gene loci in IEC that encode cell junction proteins needs to be ensured at all times. Aberrant transcriptional regulation of barrier-related genes has been reported in patients with inflammatory bowel diseases (IBD), such as ulcerative colitis and Crohn’s disease (reviewed in refs. [1, 2]). Indeed, the resulting intestinal barrier breakdown entails fatal immune activation due to facilitated translocation of microbiota and inflammatory microbial constituents across the epithelial layer. Previous studies have addressed the transcriptional regulation of cell junction proteins by transcription factors (e.g., *β-Catenin–TCF/LEF*, *HNF-4a*, *Cdx2, Cdx1, GATA4*, *Slug* and *Snail*) [3–6] as well as the epigenetic regulation of promoter chromatin accessibility via histone modifications in some detail [7]. However, our knowledge regarding the transcriptional regulation of cell junction proteins by the transcript elongation machinery promoting and fine-tuning RNA polymerase II (PolII) activity is limited. Negative elongation factor (NELF) together with DRB sensitivity-inducing factor (DSIF) are both thought to prevent transcription elongation of PolII-transcribed gene loci [8] based on the so-called promoter-proximal pause/release concept [9]. However, current findings imply that NELF, within early elongation complexes, affects transcription independent of the RNA Pol II pause/release model [10].

**Fig. 1:**
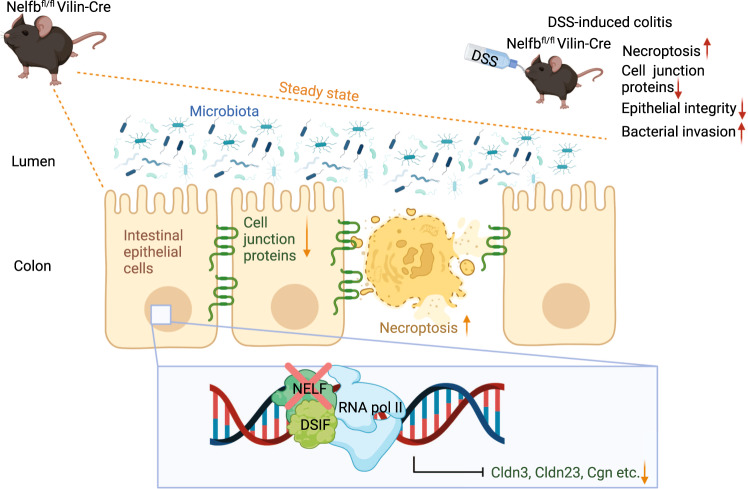
Impact of epithelial NELF on transcriptional regulation of cell junction proteins in murine IEC. Epithelial NELF ensure sufficient expression of the cell junction proteins *Cldn3*, *Cldn23* and *Cgn*. IEC-specific NELF-deficiency compromises the expression of *Cldn3*, *Cldn23,* and *Cgn* and promotes IEC necroptosis without obvious effects on intestinal barrier integrity. DSS-induced intestinal inflammation combined with IEC-specific NELF-deficiency, however, exacerbates intestinal inflammation by enhancing impairment of cell junction protein expression, IEC necroptosis, and consequently bacterial translocation from the intestinal lumen into the deeper gut tissue. Protein structures are symbolic. (Created with BioRender.com)

In a paper recently published in *Mucosal Immunology*, Ou and colleagues highlighted novel insights into the in vivo relevance of NELF for intestinal epithelial junction protein expression. The authors convincingly demonstrated a pivotal role of NELF in the maintenance of intestinal epithelial integrity in the steady state and during dextran sodium sulfate (DSS)-induced colitis by acting as a transcriptional guarantor of cell junction gene expression and preventing intestinal epithelial necroptosis [11] (Fig. 1).

Originally, NELF was identified as a protein complex consisting of 5 polypeptide subunits (NELF A to E) [8]. Loss of any of these subunits renders the entire NELF complex unstable, resulting in decreased protein abundance of all NELF constituents [12]. Although NELF was initially described as a molecular element inhibiting transcription elongation [8], debate is ongoing regarding whether this is indeed the whole story. In fact, NELF seems to have a more versatile impact on transcription than originally conceived, a conclusion driven by conflicting observations showing that a lack of NELF can result in both increased and decreased transcription [13, 14] of context-dependent NELF target genes.

Utilizing conditional knockout mice that lack the NELF-B subunit specifically in IEC, Ou et al. demonstrated that under steady-state conditions, intestinal NELF-deficiency did not result in any apparent developmental defects, histological abnormalities or altered IEC proliferation compared to wild-type controls. Strikingly, upon challenge with DSS to induce barrier breakdown, NELF-deficiency in IEC resulted in a marked worsening of intestinal inflammation, as indicated by significantly increased colon shortening, body weight loss, and mortality compared to NELF-competent mice. These results highlight a crucial contribution of the NELF complex in the maintenance of intestinal homeostasis. Intriguingly, according to body weight loss, the protective effect of IEC-expressed NELF against devastating colitis development became evident as early as day 2 after DSS treatment initiation and remained observable over the entire duration of the experiment, i.e., 12 days. Consistent with intestinal damage, deficiency in epithelial NELF resulted in increased necroptosis-like cell death in colonic IEC by day 2 of DSS treatment, which was reversible by antibiotic treatment. Thus, intestinal microbiota were identified as potential drivers of IEC necroptosis in mice deficient in epithelial NELF. Further in-depth molecular analyses by transmission electron microscopy and immunoblotting revealed that despite the absence of obvious histopathological indicators, NELF-deficient IEC undergo necroptosis-like cell death even in the steady state without compromising intestinal permeability. Evidence for the in vivo relevance of IEC necroptosis for intestinal barrier dysfunction in IEC-specific NELF-deficient mice was demonstrated by therapeutic treatment of mice with the necroptosis inhibitor necrostatin-1 during DSS treatment, ameliorating body weight loss, histology scores and intestinal damage.

Based on RNA-Seq analysis of total colon tissue, the authors specified *de facto* transcriptional consequences of IEC NELF-deficiency and found, among others, enhanced expression of goblet-cell-related transcripts (*Pnliprp2, Retnlb, Ang4, Itln1, Reg4, Ptpro, Mptx1, Spink4, Dkkl1)* as well as depressed expression of typical enterocyte-related genes *(Tgm3, Slc37a2, Fabp6, Cyp3a44, Slc6a4, Edn1, Vstm5, Pbld1, Fndc5, Atp12a*). Given that the numbers of intestinal goblet cells were not affected by NELF deficiency and only marginal alterations in microbiota composition were found, the authors attributed the observed transcriptional changes to NELF deficiency in IECs. In more detail, using positional mapping of NELF complex binding within IEC chromatin using NELF-E ChIP-Seq analysis, the authors identified NELF positioning on mouse IEC DNA to be overrepresented at gene bodies encoding epithelial junction proteins. Consistent with this finding, the protein levels of Claudin 3 (Cldn3), Cingulin (Cgn, and Claudin 23 (Cldn23), whoose gene loci also showed NELF binding, were reduced in IEC-specific NELF-deficient mice. NELFb knockdown in the human IEC cell line LS174T confirmed this observation in a different experimental setting.

The competence of NELF to ensure the expression of intestinal epithelial junction proteins under in vivo inflammatory conditions was ultimately confirmed in DSS-treated IEC-specific NELF-deficient mice, where mRNA expression of *Clnd3*, *Cgn,* and Cldn23 was impaired as well and accompanied by detrimental intestinal permeability, as demonstrated by FITC-dextran assay. Intestinal barrier damage, IEC necroptosis and consequently bacterial infestation of the deeper gut tissue were demonstrated by in situ detection of bacterial ribosomal RNA and antibody staining of Gram-negative *E. coli* and Gram-positive *Clostridia*. Given that fatal intestinal barrier breakdown in DSS-treated IEC-specific NELF-deficient mice can be prevented by dosage of broad-spectrum antibiotics, the authors conclude the overall observed fatal inflammatory phenotype unequivocally to be driven by microbial dissemination, but nevertheless, to be enhanced by impaired expression of epithelial junction proteins in IEC-specific NELF-deficiency.

In conclusion, the study by Ou et al. uncovered a hitherto unknown protective role of the NELF in maintaining intestinal barrier integrity by ensuring efficient expression of cell junction proteins in IEC and preventing fatal necroptosis-like cell death in murine experimental intestinal inflammation. It is tempting to hypothesize that impaired expression or instability of the NELF complex in humans may render the individual more susceptible to barrier breakdown and might thus be considered a cornerstone for the development of IBD once the system is challenged, e.g., by intestinal infection or any other inflammatory insult. Further studies on patient-derived intestinal biopsies are certainly needed to refute or validate this hypothesis. In the latter case, pharmacologic targeting of the NELF complex might represent a promising therapeutic approach to sustainably restore intestinal barrier function and thus to break the vicious cycle of intestinal leakiness and microbiota-driven inflammation in IBD patients.
